# Plant essential oils against woody plant fungal pathogens: chemical composition, antifungal mechanisms, and translational challenges

**DOI:** 10.1080/15592324.2026.2733230

**Published:** 2026-09-18

**Authors:** Mehrdad Alizadeh

**Affiliations:** a Soil Science Department, University of Tehran, Tehran, Iran

**Keywords:** Essential oils, woody pathogens, antifungal mechanisms, biofungicides, sustainable disease management

## Abstract

Woody plants are threatened by fungal and oomycete pathogens that cause root and wood rots, cankers, vascular wilts, dieback, fruit decay, and other diseases. Meanwhile, intensive production systems and repeated fungicide use have raised concerns about resistance, environmental persistence, and non-target effects. Essential oils (EOs) have emerged as promising natural antimicrobial resources, yet their effectiveness depends strongly on chemical composition, pathogen susceptibility, concentration, formulation, and application strategy. This review synthesizes current evidence on EO-based management of pathogens affecting forest trees, fruit trees, woody crops, and wood materials, emphasizing the chemical and biological determinants of antifungal efficacy. Across diverse pathosystems, EO activity is associated with phenolic monoterpenes, aldehydes, monoterpene hydrocarbons, and oxygenated terpenoids, while chemotypic and geographical variation can substantially influence performance. EOs act through multiple complementary mechanisms, including disruption of fungal cell membranes and walls, ergosterol depletion, interference with sterol and cell-wall biosynthesis, mitochondrial dysfunction, oxidative stress, morphological damage, inhibition of spore germination, and suppression of pathogen development. Some EOs may additionally stimulate host defense responses, indicating that disease suppression can involve both direct antifungal activity and plant-mediated resistance. Advances in vapor delivery, coatings, nanoemulsions, and wood-preservative applications further enhance their potential. However, practical deployment remains limited by compositional variability, volatility, phytotoxicity, formulation instability, limited persistence, inconsistent dose responses, and insufficient field validation. Future research should prioritize standardization, mechanism-guided formulation, synergistic combinations, ecotoxicological assessment, techno-economic evaluation, and field- and commercial-scale validation to enable reliable integration of EOs into sustainable woody plant disease management.

## 1. Introduction

Plants, particularly trees, are fundamental components of terrestrial ecosystems, contributing substantially to oxygen production, carbon sequestration, habitat formation, and climate regulation through their extensive canopies. They also play critical roles in nutrient cycling and soil stabilization while supporting biodiversity, ecosystem resilience, and human livelihoods.^1^ ^,^ ^2^ However, forest and woody plant ecosystems are increasingly exposed to multiple anthropogenic and environmental pressures, including deforestation, land-use change, drought, and pest outbreaks, which can substantially compromise their ecological stability, resilience, and functioning.^3^ ^,^ ^4^ Among these threats, plant diseases represent a major constraint to the health, productivity, and sustainability of woody ecosystems. Trees growing in natural forests, as well as those cultivated in plantations comprising native or introduced species, are increasingly threatened by fungal diseases.^2^ ^,^ ^5^ Although these diseases have traditionally been addressed within the field of forest pathology, comparatively limited attention has been given to diseases affecting woody plants beyond conventional forestry systems. Fruit trees, for instance, are rarely considered within the broader context of forest pathology, despite their evolutionary origins in natural forest ecosystems and their susceptibility to pathogens that have co-evolved with their native hosts.^6^ ^,^ ^7^ Consequently, strengthening the capacity to prevent and manage diseases while maintaining the health, productivity, and sustainable production of forest and other woody plants remains a major challenge for the long-term sustainability of the forestry sector.^8^

A diverse range of fungal and oomycete pathogens is responsible for diseases affecting woody plants. Well-known fungal pathogens, including *Heterobasidion annosum*, *Armillaria mellea*, *Fusarium solani*, *Rosellinia necatrix*, *Alternaria*, and *Puccinia*, together with oomycetes such as *Phytophthora*, *Peronospora*, and *Pythium*, cause a broad spectrum of economically and ecologically important diseases. These include downy and powdery mildews, rusts, smuts, root and wood rots, fruit rots, leaf and twig blights, stem and branch cankers, leaf spots and necrotic patches, anthracnoses, and vascular wilts.^9–17^ The increasing intensification of monoculture systems may further favor pathogen establishment and disease development by reducing biodiversity and ecosystem stability. At the same time, the widespread and repeated use of synthetic fungicides has contributed to the emergence and selection of resistant pathogen populations while raising substantial environmental and health concerns.^18–22^ Many conventional fungicides, including benzimidazoles and sterol biosynthesis inhibitors, also exhibit poor biodegradability and may pose toxicity risks to humans, beneficial microorganisms, and pollinators.^23^ ^,^ ^24^ These limitations highlight the need to diversify disease-management strategies and identify effective alternatives that combine antifungal efficacy with improved environmental compatibility.

In response to these challenges, the European Directive 2009/128/EC encourages the adoption of sustainable alternatives, including biocontrol strategies. Biopesticides can be derived from beneficial microorganisms, such as plant growth-promoting rhizobacteria (PGPR) and mycorrhizal fungi, as well as from natural substances obtained from plants, algae, and microorganisms. Among these alternatives, essential oils (EOs), which are volatile secondary metabolites synthesized by aromatic plants, have attracted increasing attention because of their antifungal properties, low mammalian toxicity, and environmental biodegradability.^25–28^ EOs are most commonly produced through steam distillation, whereas supercritical CO₂ extraction represents an alternative approach that can better preserve volatile compounds, although it generally involves higher operational costs.^29–34^ They are also classified as Generally Recognized As Safe (GRAS) by the U.S. Food and Drug Administration, further supporting interest in their potential applications as natural disease-management agents.

**Figure 1.**
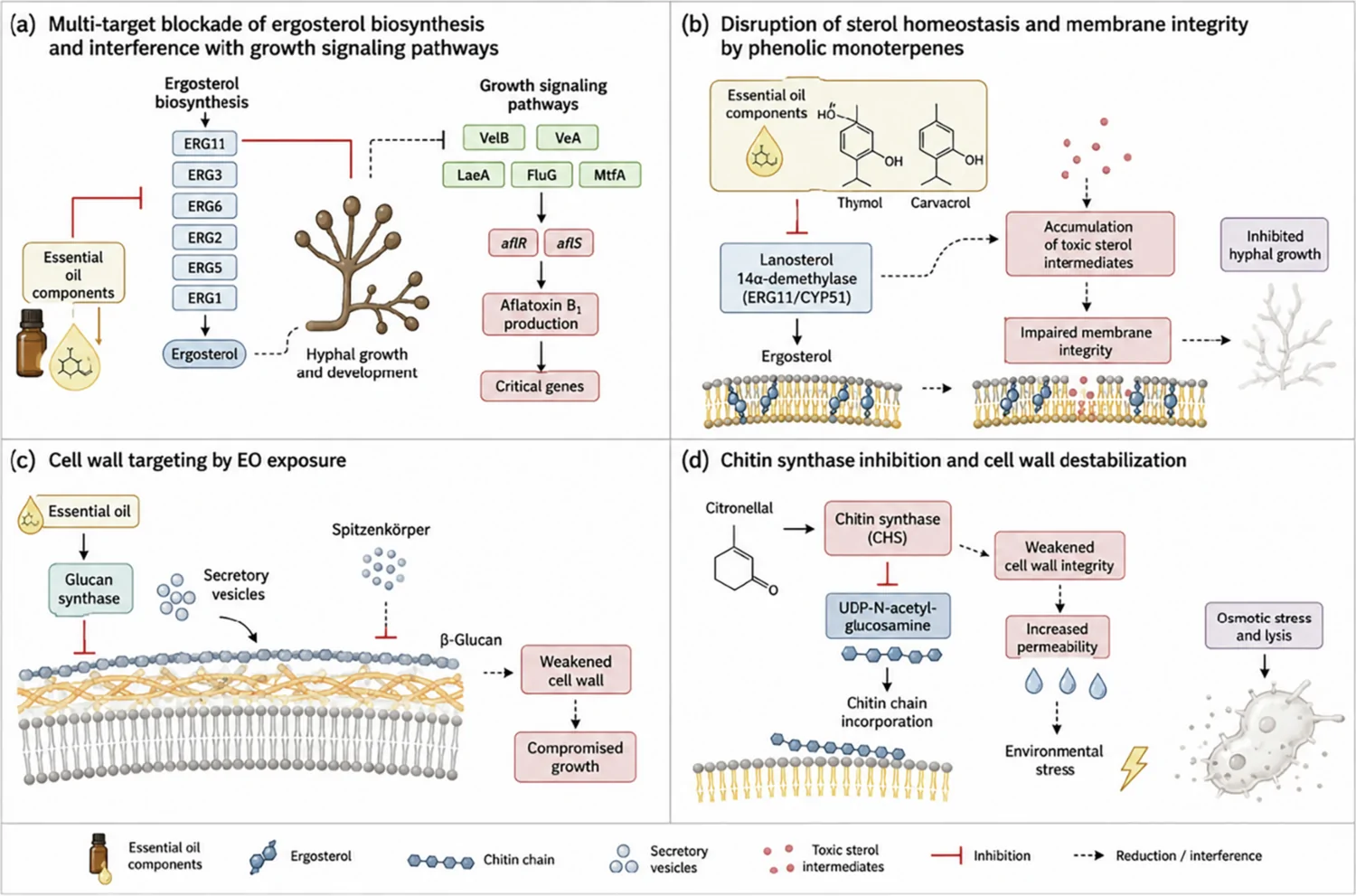
Proposed antifungal mechanisms of plant essential oils against fungal pathogens. Essential oils (EOs) exert antifungal effects through multiple, interconnected cellular targets. (a) EOs interfere with ergosterol biosynthesis and fungal growth-signaling pathways, thereby impairing membrane homeostasis, hyphal development, and secondary metabolite production. (b) Phenolic components can disrupt sterol metabolism, promote the accumulation of toxic sterol intermediates, and compromise plasma membrane integrity. (c) EO exposure interferes with glucan synthesis and secretory processes at the hyphal tip, resulting in reduced *β*-glucan deposition and weakening of the fungal cell wall. (d) Inhibition of chitin synthesis further destabilizes the cell wall and increases cellular permeability, promoting osmotic stress and lysis. Thereby, these mechanisms highlight the multitarget antifungal potential of EOs through simultaneous disruption of membrane function, cell-wall biosynthesis, cellular signaling, and structural integrity.

**Figure 2.**
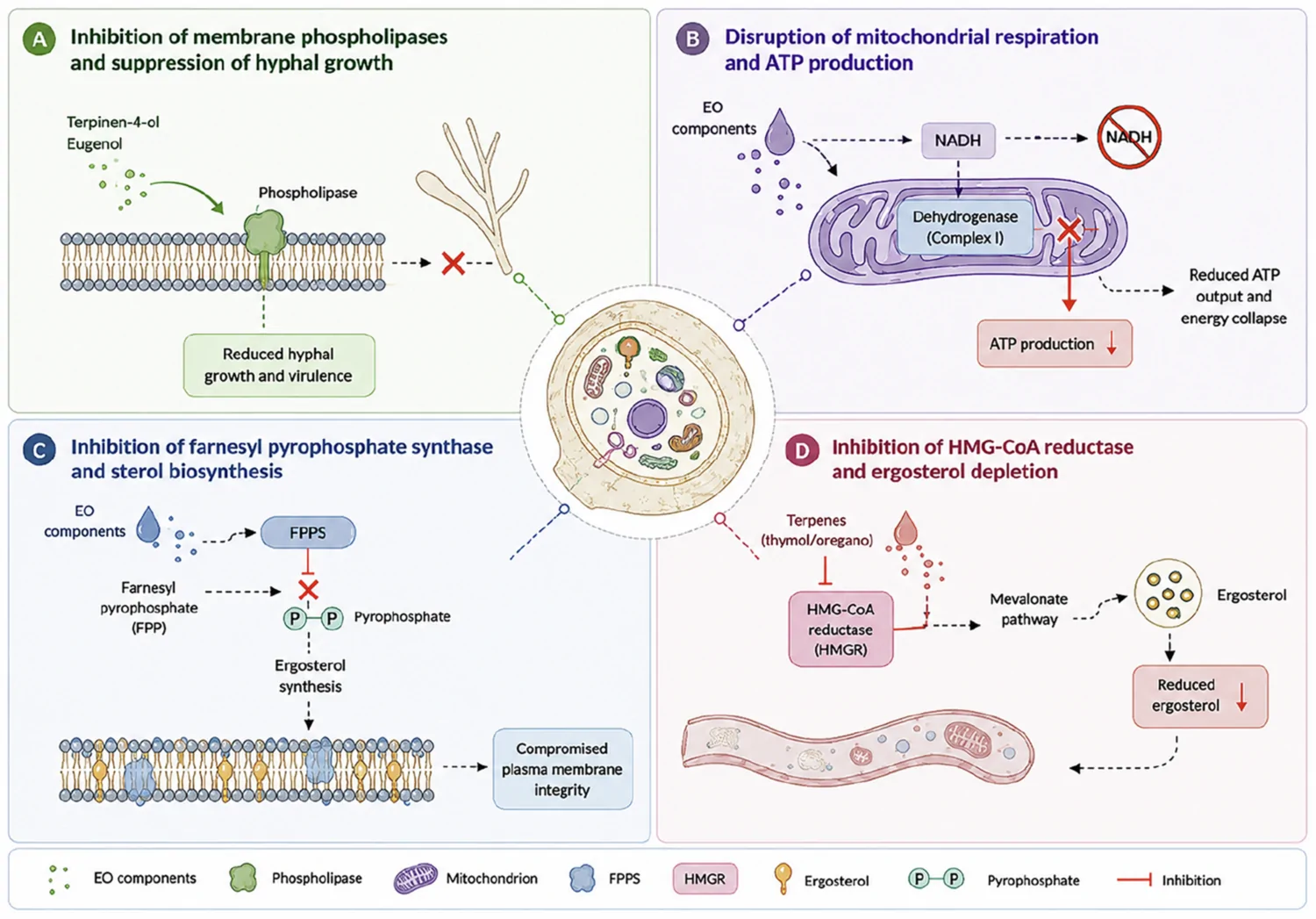
Multifaceted antifungal mechanisms of plant-derived essential oils (EOs). (a) Monoterpenoids such as terpinen-4-ol and eugenol interact with membrane-associated fungal phospholipases, disrupting phospholipid turnover and weakening cellular structures, thereby restricting hyphal extension and reducing virulence. (b) EO constituents interfere with mitochondrial NADH dehydrogenase (Complex I), disrupting electron transfer to ubiquinone, diminishing the proton-motive force, and consequently reducing ATP production. (c) Inhibition of farnesyl pyrophosphate synthase (FPPS) decreases the intracellular pool of farnesyl pyrophosphate, limiting downstream ergosterol biosynthesis and compromising plasma-membrane integrity. (d) Terpene-rich EOs from thyme and oregano inhibit HMG-CoA reductase (HMGR), the rate-limiting enzyme of the mevalonate pathway, thereby reducing mevalonate flux and further depleting cellular ergosterol. Collectively, these complementary mechanisms disrupt fungal lipid metabolism and respiratory energy production, ultimately suppressing fungal growth and viability.

The biological activity of EOs is closely associated with their complex chemical composition. These mixtures are predominantly composed of terpenes, including monoterpenes such as *p*-cymene, limonene, and *α*- and *β*-pinene, together with oxygenated terpenoids such as carvacrol, thymol, and camphor (Figure 1).^35–39^ Importantly, EO composition is highly variable and can be influenced by plant species, genotype, environmental conditions, developmental or maturity stage, and extraction method.^40^ ^,^ ^41^ Such chemical variability is particularly relevant to their antifungal performance because the biological activity of an EO is determined not only by the presence of individual compounds but also by their relative abundance and interactions within the complex mixture. Owing to their lipophilic nature and low molecular weight, terpene constituents can interact directly with fungal cells, disrupting membrane integrity and affecting essential cellular processes. Reported effects include inhibition of sporulation and spore germination as well as interference with ergosterol biosynthesis, mitochondrial respiration, and nucleic acid synthesis (Figure 2).^42–44^ Several individual EO constituents, including 1,8-cineole, limonene, and *p*-cymene from *Eucalyptus* spp.; carvacrol and thymol from *Origanum vulgare* and *Thymus vulgaris*; citral and linalool from citrus leaves; and compounds derived from lavender, rosemary, and other Lamiaceae species, have demonstrated significant antifungal activity against agriculturally important pathogens, including *Fusarium*, *Botrytis cinerea*, *Penicillium*, *Aspergillus*, *Phytophthora*, and *Monilinia.* ^45–48^ The primary objective of this review is to critically evaluate the potential of essential oils for sustainable management of fungal and oomycete pathogens affecting woody plants, encompassing forest trees, fruit trees, woody crops, and wood materials. Specifically, this review addresses four guiding questions: (1) What are the chemical determinants of essential oil antifungal activity against woody pathogens, and how does chemotypic variation influence efficacy? (2) What are the molecular, cellular, and physiological mechanisms by which essential oils suppress woody fungal pathogens, and how do these mechanisms differ across pathogen groups? (3) What factors govern the translation of in vitro activity to in vivo and field efficacy, including formulation, application strategy, phytotoxicity, and environmental influences? (4) What emerging technologies and approaches—including nanotechnology, precision agriculture, and systems biology—could address current limitations and enable practical deployment? The review is structured around a conceptual framework that progresses from chemical composition through mechanistic understanding to applied challenges and future opportunities, with the goal of providing a critical synthesis that informs both research priorities and practical disease management strategies for woody plant systems.

Despite growing interest in essential oils as biofungicides, existing reviews have predominantly focused on agricultural crops and herbaceous plants, with limited systematic attention to woody plant pathosystems. This gap is significant because woody pathogens differ fundamentally from those affecting annual crops: they cause chronic infections that persist across multiple seasons, colonize vascular tissues and wood substrates, and require management strategies that address long-term disease progression rather than acute outbreaks. This long-term perspective also requires consideration of whether EO-based treatments can be delivered repeatedly and economically at orchard, plantation, forest, and commercial postharvest scales, rather than relying primarily on laboratory-scale efficacy or small-volume postharvest treatments. Furthermore, woody plants represent distinct ecological and economic contexts, encompassing forest trees, fruit orchards, plantation species, and timber products, each with unique disease pressures and management constraints. While previous reviews have addressed essential oil applications in postharvest fruit preservation or general agricultural contexts, none has comprehensively synthesized the evidence specifically for woody fungal pathogens across these diverse production systems. The present review addresses this gap by systematically evaluating the antifungal potential of essential oils against pathogens of woody plants, with particular emphasis on the chemical drivers of activity, mechanistic pathways, and the translational challenges that distinguish woody pathosystems from other agricultural contexts.

## 2. Literature search and study selection

This review was conducted through systematic literature searches in the following electronic databases: Web of Science, Scopus, PubMed, Google Scholar, and CAB Abstracts. The search covered publications from January 2000 through June 2026, reflecting the period during which essential oil research for woody plant pathogen management has substantially expanded. Primary search terms included combinations of the following keywords: “essential oils,” “plant essential oils,” “antifungal,” “woody pathogens,” “tree pathogens,” “forest pathogens,” “fruit tree diseases,” “wood decay fungi,” “postharvest fungi,” “mechanism of action,” “biofungicide,” and “sustainable disease management.” Additional targeted searches were conducted for specific pathogen genera (*Botryosphaeria*, *Colletotrichum*, *Fusarium*, *Phytophthora*, *Monilinia*, *Neofusicoccum*), plant hosts (fruit trees, forest trees, woody crops), and oil sources (*Thymus*, *Origanum*, *Cinnamomum*, *Eucalyptus*, *Syzygium*). Reference lists of retrieved articles and relevant reviews were manually screened to identify additional studies not captured through database searches. Studies were included if they reported original experimental data on essential oil antifungal activity against pathogens of woody plants, including in vitro assays, in vivo experiments, and field trials.

## 3. EOs as Biofungicides

Growing evidence indicates that certain chemical fungicides impose severe, long-term adverse effects on both human health and the environment, making exclusive reliance on synthetic pesticides for plant disease management increasingly undesirable. Essential oil (EO)-based biofungicides offer a more environmentally benign alternative, particularly against phytopathogenic fungi.^49^ Considerable in vitro research has been devoted to expanding our understanding of EOs' fungicidal activity, with fungal strains either obtained from pathology collections or isolated from contaminated substrates such as infected plant tissues and heritage objects.^50^ ^,^ ^51^ Multiple studies have highlighted the potential of EOs to reduce postharvest fruit losses.^52–55^ For example, at concentrations of 500–1000 µL L^−1^, several EOs effectively prevented citrus decay caused by *Geotrichum citri-aurantii*, although they acted only fungistatically and did not eradicate the fungal colonies entirely.^55^ In contrast, other investigations found that EOs exhibit stronger antifungal effects in the vapor phase.^53^ ^,^ ^54^ Vapors of *Thymus vulgaris* and *Cinnamomum verum* EOs (16.7 µL L^−1^) controlled brown rot in peaches caused by *Monilinia laxa.* ^56^ The EO of *Origanum vulgare* also demonstrated potent, broad-spectrum antifungal activity against *Monilinia fructicola*, *Aspergillus niger*, *Penicillium expansum*, and *B. cinerea*, especially at 500 and 1000 mg L^−1.^ ^57^ For example, the essential oil from *Cicuta virosa*, characterized by high proportions of *γ*-terpinene (40.92%), *p*-cymene (27.93%), and cumin aldehyde (21.20%), exhibited strong inhibitory effects on spore germination and completely suppressed aflatoxin B1 production in *Aspergillus flavus* at 4 µL/mL, illustrating the chemical diversity underlying EO bioactivity.^58^ Also, *Litsea cubeba* essential oil (LCO), composed mainly of trans-citral, cis-citral, and d-limonene, has also been reported to exhibit antifungal activity against *Botrytis cinerea*, a major postharvest pathogen responsible for the decay of fruits and vegetables.^59^ ^,^ ^60^ This efficacy is attributed to the lipophilic phenolic compounds carvacrol and thymol, which disrupt fungal cells by interfering with protein synthesis, energy metabolism, and membrane integrity.^61^ Similarly, EOs from six *Satureja* species—whether applied in vapor phase or incorporated into growth medium—effectively inhibited the growth of nine *Penicillium* species that commonly infect citrus fruits, at 10, 20, and 30 µL concentrations.^62^ Additionally, *Eucalyptus cinerea* and *E. nicholii* EOs (10 µL in ethanol) proved effective against *B. cinerea* and *F. oxysporum* f.sp. *lycopersici.* ^63^ EOs from *Pinus pinea* and *Pinus nigra* (4 µL mL^−1^) exhibited antifungal activity against *B. cinerea*, *Bipolaris sorokiniana*, *Fusarium culmorum*, *F. graminearum*, and *F. avenaceum*, with limonene, *α*-pinene, and *β*-pinene as major components.^64^ The oil of *C. sempervirens* was likewise reported to inhibit a range of plant-pathogenic fungi.^65^

## 4. EOs effects on woody pathogenic fungi

EOs have demonstrated considerable potential for the management of woody plant and postharvest fungal pathogens, with their antifungal efficacy varying according to plant source, chemical composition, target pathogen, and mode of application (Tables 1a–d). In one of the early studies, leaf essential oils of *Cinnamomum osmophloeum* collected from different provenances were classified into six chemotypes based on GC–MS profiling and cluster analysis, and the cinnamaldehyde and cinnamaldehyde–cinnamyl acetate chemotypes exhibited strong inhibitory activity against four tree pathogenic fungi; among 14 identified constituents, Z-cinnamaldehyde, eugenol, geraniol, and citral showed the highest activity, with Z-cinnamaldehyde being the most potent compound.^66^ Similarly, essential oils from five Moroccan plants were evaluated against *Fusarium oxysporum* f. sp. *albedinis*, the causal agent of Bayoud disease in date palm, and *Origanum compactum* (MFC 5 µL/mL) and *Thymus satureioides* (MFC 13 µL/mL) demonstrated substantial antifungal activity, with thymol and carvacrol identified as particularly potent constituents (MFC 0.94 and 2.08 µL/mL, respectively), highlighting the importance of phenolic monoterpenes in EO-mediated fungal inhibition and their potential as eco-friendly alternatives to synthetic fungicides.^67^ Beyond direct antifungal activity, EOs may also act through stimulation of plant defense responses, as demonstrated by fumigation of grapevines with *Origanum vulgare* EO for 24 h after infection, which reduced *Plasmopara viticola* infection by 95%; transcriptomic analysis revealed less than 1% pathogen-derived reads in treated plants compared with approximately 30% in controls, while activation of salicylic acid-, jasmonic acid-, and ethylene-dependent pathways, together with induction of pathogenesis-related proteins and phytoalexins, indicated that the protective effect was largely associated with activation of host innate immunity rather than direct pathogen killing.^68^ The potential of EOs for protecting woody materials has also been demonstrated against wood-decay fungi. Essential oils obtained from 18 Egyptian plants were hydrodistilled, characterized by GC–MS, and evaluated against *Hexagonia apiaria* and *Ganoderma lucidum*, with *Artemisia monosperma* exhibiting the strongest activity, with EC₅₀ values of 31 and 53 mg/L, respectively; six highly active oils significantly reduced mass loss in Scots pine sapwood, with *Citrus limon* showing particular efficacy against *H. apiaria* and *A. monosperma* against *G. lucidum*, supporting their potential application in wood preservation.^69^ EO activity has likewise been reported against important postharvest pathogens associated with woody and fruit crops. Five EOs, including thyme, clove, cinnamon, anise, and vitex oils, were tested against *Colletotrichum gloeosporioides* and *Lasiodiplodia theobromae*, major postharvest pathogens of mango, and thyme EO at 5 µL and clove and cinnamon EOs at 8 µL per plate produced complete in vitro inhibition; moreover, thyme EO, characterized by thymol, o-cymol, and terpinolene, significantly suppressed pathogen development when applied as a fumigant at 66.7 µL/L, demonstrating its potential as a natural postharvest fungicide.^54^ Dill seed essential oil, rich in apiole, carvone, dihydrocarvone, and limonene, also showed dose-dependent inhibition of *Neofusicoccum parvum*, the causal agent of postharvest chestnut rot, through disruption of cell wall and membrane integrity, induction of oxidative stress, and modulation of genes associated with replication and lipid metabolism; in vivo application further prolonged chestnut storage, indicating its potential as a natural preservative.^70^ Consistent with these findings, four Piperaceae EOs were evaluated against *Fusarium solani* isolated from mango and avocado, with *Piper lanceaefolium* EO producing the highest inhibition (69.6% at 300 ppm), while limonene and *α*-pinene contributed substantially to the antifungal activity, with inhibition values of 78% and 74%, respectively, although considerable variation in EO composition and efficacy was observed among species.^71^ Recent investigations have further confirmed the broad-spectrum potential of EOs against pathogens affecting olive trees. Six commercial EOs tested against six olive tree pathogens were dominated by monoterpenoids, except clove and garlic oils, which were characterized mainly by eugenol and trisulfide, respectively; although tea tree, rosemary, thyme, and oregano oils showed limited activity at lower concentrations, clove EO exhibited broad-spectrum inhibition against *Fusarium* spp., *Verticillium dahliae*, and *Lasiodiplodia theobromae* at 500–4000 ppm, while *Biscogniauxia mediterranea* was inhibited by clove, garlic, and rosemary oils at 4000 ppm, making clove EO the most promising candidate among the tested oils.^72^ Mechanistic evidence has also emerged from studies of wood-derived EOs, with *Phoebe bournei* wood essential oil showing strong activity against *Botryosphaeria dothidea* and effectively controlling apple ring rot by inducing morphological and membrane damage, increasing extracellular conductivity and the leakage of nucleic acids and proteins, reducing ergosterol and trehalose levels, inducing reactive oxygen species and mitochondrial dysfunction, and altering the expression of genes involved in fungal cell-wall and membrane biosynthesis, including upregulation of GH16 and downregulation of CHS, FKS, and ERG28; (-)-borneol, isoborneol, terpinen-4-ol, guiazulene, and o-cymene were identified among its active constituents, supporting its potential for postharvest apple protection.^73^ Likewise, six Tunisian EOs were screened against *Neofusicoccum parvum*, an important grapevine trunk disease pathogen, and *Thymus capitatus* EO, containing 68.84% carvacrol, showed the greatest efficacy, achieving complete inhibition at 20 µL, with MIC and MFC values of 250 and 900 ppm, respectively, whereas pure carvacrol was even more potent, with MIC and MFC values of 80 and 200 ppm, emphasizing the contribution of major phenolic constituents to EO activity.^74^ Advances in formulation technology have further improved the applicability of EOs, as demonstrated by seven EO-based nanoemulsions evaluated against the citrus pathogens *Colletotrichum gloeosporioides* and *Neofusicoccum parvum*; clove- and garlic-based nanoemulsions produced the strongest in vitro inhibition based on EC₅₀ and EC₉₀ values and significantly reduced lesion development in vivo on lemon fruits and twigs, whereas lavender nanoemulsion showed limited in vivo efficacy despite promising in vitro activity, and phytotoxicity was observed at higher concentrations, emphasizing the need for optimization and field validation.^75^ More recently, *Cymbopogon martinii* EO was evaluated against *C. gloeosporioides* and *Fusarium longifundum*, achieving complete inhibition at 1200–1500 µg/mL through disruption of fungal membranes, including reduced ergosterol content, cellular leakage, acidification, and ultrastructural damage; although its activity was lower than that of Bavistin, its composition dominated by oxygenated monoterpenes and its strong antifungal activity support its potential as a natural agent for sustainable postharvest papaya preservation.^76^ So, these findings demonstrate that EOs can suppress woody and postharvest pathogens through multiple complementary mechanisms, including disruption of cell membranes and walls, leakage of intracellular components, inhibition of enzymes and essential biosynthetic pathways, oxidative and mitochondrial stress, inhibition of spore germination and mycelial growth, and, in some cases, activation of host defense signaling; however, their efficacy is strongly dependent on chemical composition, pathogen sensitivity, concentration, formulation, and application strategy, indicating that standardized EO formulations and further in vivo and field-scale validation are essential for their practical integration into sustainable disease-management programs.

### 4.1. Comparative analysis of in vitro, in vivo, and field efficacy

A critical pattern emerging from the synthesized literature is the substantial disparity between in vitro efficacy and performance in plant-based systems or field conditions. Across the studies reviewed, complete mycelial inhibition in agar-based assays was frequently achieved at essential oil concentrations of 500–2000 ppm, yet equivalent in vivo efficacy typically required higher concentrations, more frequent applications, or advanced formulations. For example, clove and thyme oils that completely inhibited *Colletotrichum gloeosporioides* at 5–8 µL/plate in vitro required fumigation at 66.7 µL/L to achieve significant postharvest protection on mango.^54^ Similarly, *Origanum vulgare* oil demonstrated potent direct antifungal activity in vitro, but its protective effect against grapevine downy mildew was attributed primarily to host defense priming rather than direct pathogen inhibition, with 95% disease reduction achieved through a 24-hour vapor exposure that elicited systemic resistance pathways.^68^ This divergence highlights the importance of host physiology, tissue architecture, and environmental factors in mediating EO efficacy.

Dose-dependent phytotoxicity represents a further constraint on in vivo translation. Lopez-Reyes et al.^77^ demonstrated that while 1% oregano, savory, and thyme oils effectively controlled *Monilinia laxa* and *Botrytis cinerea* on apricots and plums, the same concentrations caused significant phytotoxic damage on nectarines, emphasizing that fruit cultivar, surface properties, and wax composition influence sensitivity. La Quatra et al.^75^ similarly observed phytotoxic effects at higher nanoemulsion concentrations on citrus tissues, limiting the applicable dose range despite promising in vitro activity. These findings suggest that phytotoxicity thresholds are pathogen-dependent, host-dependent, and formulation-dependent, requiring empirical optimization for each pathosystem.

Chemotype-related variability further complicates translational efforts. Studies on *Cinnamomum osmophloeum,* ^66^ *Eugenia uniflora,* ^78^ and *Corymbia citriodora* ^79^ all demonstrated that oils from the same species, but different chemotypes, exhibited dramatically different antifungal efficacy. The cinnamaldehyde chemotype of *C. osmophloeum* was highly active against tree pathogens, whereas the camphor chemotype was largely ineffective. This variability underscores the necessity of chemical standardization and chemotype selection for reproducible disease management.

Formulation and application strategy constitute the most promising avenues for bridging the in vitro-in vivo gap. Nanoemulsions, as demonstrated by La Quatra et al.,^75^ improved the aqueous dispersibility and bioavailability of essential oils, with clove and garlic nanoemulsions showing enhanced in vivo efficacy against citrus pathogens compared with free oils. Vapor-phase applications^54^ ^,^ ^80^ ^,^ ^81^ offer another strategy, minimizing phytotoxicity while enabling uniform exposure in enclosed systems, though persistence and field applicability remain challenging. Controlled-release systems, including coatings^82^ and encapsulation technologies, represent further advances that may improve persistence and reduce effective doses. So, these obsoervations indicate that the translation of essential oil activity from laboratory to field requires not only selection of potent oils but also systematic optimization of formulation, dose, application timing, and delivery method tailored to specific host-pathogen-environment interactions.

## 4.2. Resistance Risk, Economic Feasibility, and Field-Scale Scalability

The long-term deployment of EOs requires careful consideration of pathogen adaptation and the potential development of reduced sensitivity. The chemically diverse and multitarget nature of EOs may reduce the probability of rapid resistance compared with conventional single-site fungicides, because multiple cellular processes can be affected simultaneously, including membrane integrity, sterol metabolism, mitochondrial function, and oxidative homeostasis.^83^ ^,^ ^84^ ^,^ ^85^ However, EOs should not be considered inherently resistance-proof. Repeated exposure to sublethal concentrations can impose selection pressure, and reduced sensitivity to individual EO constituents remains biologically plausible. Although direct evidence for stable, heritable resistance to EOs in plant-pathogenic fungi is currently limited, resistance and tolerance remain insufficiently understood and warrant systematic investigation.^84^ ^,^ ^86^ It is also important to recognize that mechanistic insights derived from human pathogens, such as geraniol-induced membrane disruption and ergosterol inhibition in *Candida albicans,* ^87^ cannot be directly extrapolated to woody plant pathogens without dedicated pathosystem-specific validation, underscoring the need for caution in cross-kingdom generalization of EO mechanisms. Future studies should therefore assess changes in EC₅₀ or MIC values following serial exposure, cross-sensitivity among chemically related oils and major constituents, and potential fitness costs. Where repeated applications are required, rotation among chemically distinct EOs and integration with biological control, host resistance, cultural practices, or reduced-risk fungicides may help diversify selection pressure and improve durability, consistent with integrated disease-management principles.^26^ Beyond essential oils, biocontrol agents such as *Bacillus velezensis* have demonstrated analogous multitarget antifungal mechanisms—including cell wall disruption, oxidative stress induction, and mitochondrial dysfunction—against the woody pathogen *Ceratocystis fimbriata,* ^88^ suggesting that integrating EOs with microbial biocontrol could diversify selection pressure and improve the durability of resistance management strategies.

Economic feasibility represents an equally important constraint because biological efficacy does not necessarily translate into commercial viability. The cost of EO-based disease management is influenced by raw-material availability, oil yield, extraction energy, chemical characterization and standardization, formulation, storage, transport, application rate, labor, and treatment frequency. Importantly, plant-derived products should not be assumed to be intrinsically cheaper than synthetic fungicides; techno-economic analyzes indicate that essential-oil production costs can vary substantially with extraction technology, energy consumption, yield, and operating requirements.^89^ Formulation can improve the stability, dispersibility, and persistence of volatile EO constituents, but encapsulation, controlled-release systems, and other advanced formulations may introduce additional manufacturing costs and technological complexity.^84^ ^,^ ^90^ Consequently, commercial development should evaluate cost per treated area, treatment frequency, formulation stability, labor and machinery requirements, and the economic value of disease suppression rather than antifungal efficacy alone.

Field-scale scalability is particularly challenging in woody systems because orchards, nurseries, plantations, and mature forests differ markedly in canopy structure, accessibility, pathogen distribution, and treatment requirements. Evidence from woody plants demonstrates that EO efficacy can extend beyond laboratory assays; for example, thyme EO reduced disease development and improved crown condition in *Fraxinus angustifolia* seedlings challenged with *Hymenoscyphus fraxineus.* ^91^ Nevertheless, seedling-scale evidence cannot yet be extrapolated directly to mature forest stands. Targeted applications in nurseries, young plantations, high-value trees, localized disease foci, pruning wounds, or postharvest materials may therefore be more immediately feasible than uniform treatment of extensive forest areas. This distinction is important because the current evidence base remains dominated by laboratory, greenhouse, seedling, and localized studies rather than replicated, multi-season commercial-scale trials. Thus, the major translational challenge is not simply demonstrating biological activity but achieving consistent, economically viable delivery at the scale required for a particular woody production system.

Thereby, the most realistic pathway is unlikely to be complete replacement of synthetic fungicides by EOs. Instead, EOs may provide greater value as standardized, formulation-specific components of integrated disease management, particularly when locally available raw materials, optimized formulations, targeted delivery, and reduced treatment frequency improve cost-effectiveness. Future research should combine multi-location and multi-season field trials with techno-economic analyzes reporting EO yield and composition, formulation and application costs, dose, treatment frequency, disease reduction, persistence, and operational requirements. Such evidence is essential for determining whether EO-based technologies can progress from promising experimental treatments to economically viable and operationally scalable tools for orchard and forestry disease management.

## 5. Mechanistic basis of essential oil antifungal activity

Plant essential oils exert antifungal activity through multiple interconnected cellular and molecular mechanisms, rather than through a single mode of action. Their effects range from disruption of fungal membranes and cell-wall integrity to inhibition of essential metabolic pathways, interference with morphogenesis and cytoskeletal organization, and induction of oxidative stress. These mechanisms can act simultaneously or sequentially, ultimately impairing fungal growth, reproduction, pathogenicity, and survival.

### 5.1. Fungal cell membrane disruption and structural damage

Disruption of the fungal cell envelope is one of the most consistently reported mechanisms underlying the fungicidal activity of EOs. Exposure to EOs can compromise cell-wall and plasma-membrane integrity, induce cytoplasmic coagulation, and cause extensive damage to intracellular organelles.^36^ ^,^ ^92^ These structural effects can ultimately impair cellular homeostasis and lead to fungal cell death. For example, clove oil markedly inhibited *Colletotrichum gloeosporioides* in sweet cherry, with scanning and transmission electron microscopy revealing severe disruption of fungal architecture, including cell-wall breakage, damage to the endomembrane system, membrane leakage, and extravasation of intracellular contents.^93^ Consistent with these observations, exposure to EOs has been associated with twisted and flattened hyphae accompanied by extensive surface debris.^94^ ^,^ ^95^ The strong lipophilicity of many EO constituents facilitates their interaction with and penetration into fungal cellular membranes. These hydrophobic compounds can interact with membrane-associated fatty acids, phospholipids, and sterols, thereby disturbing the structural organization and permeability of the fungal cell envelope.^96^ Ergosterol, a fungus-specific membrane sterol, represents an important target of this activity. Interaction with ergosterol can increase membrane permeability and impair membrane-associated functions, including ATPase-mediated H⁺ efflux, resulting in cellular swelling, hyphal disruption, and ultimately cell death.^97^ ^,^ ^98^ The resulting loss of membrane homeostasis can initiate a cascade of osmotic imbalance, ion leakage, organelle dysfunction, and cytoplasmic loss, ultimately compromising cellular viability.^99^ For example, vapor-phase application of perillaldehyde, a major constituent of perilla essential oil, effectively controlled *Aspergillus niger* decay on grapes through disruption of membrane integrity and significant depletion of ergosterol content, confirming the membrane-targeting mode of action in fruit pathosystems.^100^

Experimental evidence further demonstrates that EO-mediated membrane disruption is accompanied by substantial ionic imbalance. For instance, *Mentha cardiaca* EO induced the leakage of Ca^2^⁺, K⁺, and Mg^2^⁺ ions from *Aspergillus flavus* cells, providing direct evidence of compromised membrane integrity.^101^ Similar ultrastructural alterations have been observed in the oomycete *Phytophthora infestans* following exposure to different EOs.^102^ In *A. niger*, treatment with a EO mixture derived from *Thymus eriocalyx* and *Thymus x-porlock* resulted in severe mitochondrial rupture together with extensive damage to the cell wall and plasma membrane.^103^ Likewise, *Ageratum conyzoides* EO caused reduced polarization of mitochondrial cristae and induced rough, invaginated plasma-membrane vesicles in *A. flavus.* ^104^ Cinnamon EO exposure produced shrunken conidia, disrupted hyphae, and atypical cellular masses in *A. flavus.* ^105^ Comparable alterations in the plasmalemma and cytoplasm of *Penicillium italicum* were also observed following treatment with citral, a major constituent of several EOs.^106^ Thereby, these observations indicate that membrane damage is closely associated with broader ultrastructural deterioration involving the cell wall, plasma membrane, mitochondria, and cytoplasmic organization.

The chemical diversity of EOs contributes to their broad spectrum of biological activities, including potent antifungal effects.^107^ Among the multiple mechanisms reported, disruption of the fungal cell membrane remains one of the most extensively documented. Terpenoids, phenolic compounds, and aldehydes—including thymol, carvacrol, and citral—can interact with the fungal lipid bilayer and alter its fluidity and permeability.^108^ Increased permeability promotes the leakage of intracellular components, disrupts ionic homeostasis, and ultimately leads to collapse of membrane integrity.^109^ The hydrophobic constituents of EOs can become incorporated into the phospholipid bilayer and disturb membrane organization by interacting with essential sterols and phospholipids.^110^ In particular, thymol and carvacrol have been reported to target ergosterol, an essential determinant of fungal membrane stability, providing a mechanistic basis for their pronounced antifungal activity.^111^ Similarly, *Cinnamomum jensenianum* essential oil, rich in eucalyptol and *α*-terpineol, caused a marked reduction in ergosterol quantity and induced severe ultrastructural alterations in *Aspergillus flavus*, further supporting the role of terpenoid-rich oils in membrane-targeted antifungal activity.^112^ Beyond their direct effects on fungal structures, EOs may also enhance disease-control efficacy when combined with conventional fungicides. Such combinations can produce synergistic antifungal effects, potentially allowing lower fungicide doses while reducing the likelihood of resistance development.^84^ This complementary activity may decrease chemical inputs and environmental residues and therefore further support the integration of EO-based approaches into sustainable disease-management strategies.^113^

Importantly, membrane disruption should not be considered an isolated event but rather as an initiating process that can propagate damage throughout the fungal cell. Loss of membrane integrity can impair ion gradients and energy metabolism, promote leakage of ATP and other cytoplasmic components, and subsequently trigger mitochondrial and oxidative dysfunction. Figure 1 summarizes the proposed antifungal mechanisms of plant-derived EOs, illustrating their effects on fungal cell walls, plasma membranes, and intracellular organelles, as well as the associated cellular leakage, oxidative imbalance, and enzymatic inhibition that collectively contribute to fungal cell death.

### 5.2. Enzymatic and molecular inhibition of key fungal pathways

The antifungal activity of EOs is largely attributable to their ability to interfere with key enzymatic networks governing fungal metabolism, energy production, and cell-wall integrity (Leiva‑Mora et al. 2025). Beyond inflicting physical damage, EOs exert biochemical disruption by targeting critical enzymes involved in energy generation, sterol biosynthesis, and cell‑wall assembly. Ajwain essential oil, for instance, not only suppresses ergosterol synthesis but also downregulates the regulatory genes velB, veA, laeA, fluG, and Mtfa, along with the developmental genes brlA and abaA, in Aspergillus flavus; it further interferes with the aflatoxin activators aflR and aflS.^114^ At the cell‑wall level, various EO components inhibit key polymer‑synthesizing enzymes, including lanosterol demethylase,^115^ glucan synthase,^116^ and chitin synthase^73^—with independent corroboration of chitin synthase inhibition also reported.^117^ Investigations on dill seed essential oil revealed that its antifungal action involves simultaneous disruption of the plasma membrane permeability barrier and mitochondrial dysfunction, as evidenced by reduced ergosterol content, elevated mitochondrial membrane potential, and suppression of ATPase and dehydrogenase activities in *Aspergillus flavus.* ^118^ Blockade of these enzymes weakens cell‑wall rigidity, elevates membrane permeability, and renders fungi more susceptible to environmental challenges. Mitochondrial respiration represents another major target. Inhibition of cytochrome c oxidase (Complex IV) impairs electron transfer and ATP production, causing energy starvation and reactive oxygen species (ROS) accumulation, while NADH dehydrogenase inhibition disrupts oxidative phosphorylation, leading to metabolic imbalance and heightened oxidative stress.^117^ Oxidative injury is further exacerbated by the capacity of EOs to suppress endogenous antioxidant enzymes, notably catalase (CAT) and superoxide dismutase (SOD), which amplifies intracellular ROS levels and promotes cellular damage.^119^ Additionally, certain compounds—such as terpinen‑4‑ol and eugenol—block phospholipases, thereby compromising lipid metabolism and cellular signaling pathways.^120^ Regarding sterol biosynthesis, several terpenes directly target the mevalonate pathway at multiple junctures. They inhibit HMG‑CoA reductase (HMGR),^121^ mevalonate‑5‑pyrophosphate decarboxylase (MVD),^122^ and farnesyl pyrophosphate synthase (FPPS).^123^ At later stages, EO constituents—particularly thymol and carvacrol—disrupt membrane assembly by targeting lanosterol synthase (LSS),^124^ C‑14 sterol demethylase (CYP51),^125^ and C‑24 methyltransferase (ERG6),^126^ thereby impairing ergosterol production and compromising fungal membrane integrity (Figure 2).

### 5.3. Morphological and cytoskeletal alterations in fungi

Beyond their direct morphological effects, EOs also potently inhibit spore and conidial germination across a broad spectrum of phytopathogens—although this efficacy is strongly governed by EO composition, concentration, and the target fungal species (Leiva-Mora et al. 2025). Morphogenesis—the process governing hyphal differentiation, spore formation, and reproductive development—is fundamental to fungal pathogenicity.^127^ EOs interfere with these processes by directly targeting cytoskeletal elements, disrupting both microtubule dynamics and actin organization.^83^ Clove oil, which is abundant in eugenol, impairs microtubule assembly and stability, thereby hindering vesicle transport and cell division and ultimately suppressing fungal proliferation.^128^ Similarly, thymol disrupts actin filaments concentrated at hyphal tips by altering polymerization dynamics, which impedes vesicle trafficking and restricts hyphal elongation.^129^ Taken together, such structural disturbances compromise nutrient uptake, tissue colonization, and overall virulence.

During reproductive stages, EOs inhibit the formation and germination of conidia—the asexual spores key to fungal dispersal—thereby limiting infection cycles.^130^ Specific compounds, including thymol, carvacrol, and eugenol, reduce spore dimensions, viability, and germination efficiency, directly diminishing pathogenic potential.^131^ Tea tree and cinnamon oils further modify hyphal and mycelial architecture, suppressing aerial mycelium formation and attenuating metabolic activity.^132^ Likewise, neem (*Azadirachta indica*) and tea tree EOs have been shown to markedly inhibit urediniospore germination in *Hemileia vastatrix*, underscoring their promise as natural fungicidal agents in agricultural settings.^133^

### 5.4. Oxidative stress and lipid peroxidation as determinants of antifungal activity

A key antifungal mechanism of EOs involves the induction of oxidative stress via excessive reactive oxygen species (ROS) generation.^134^ Upon penetrating fungal membranes, phenolic and terpenoid constituents interfere with mitochondrial cytochrome c oxidase activity, causing electron leakage and the one-electron reduction of oxygen to superoxide radicals (O^2•−^).^135^ These radicals are then converted to hydrogen peroxide (H_2_O_2_) by superoxide dismutase and subsequently to hydroxyl radicals (•OH) through Fenton reactions.^136^ The resulting ROS accumulation overwhelms endogenous antioxidant defenses, inflicting oxidative injury on lipids, proteins, and nucleic acids.^137^ Thymol and carvacrol are notably potent in triggering ROS-mediated apoptosis-like pathways in *Candida albicans* and *Aspergillus niger,* ^138^ while cinnamaldehyde and eugenol similarly elevate intracellular ROS levels, promoting lipid peroxidation, mitochondrial dysfunction, and membrane disruption.^139^ Nerol, a monoterpene alcohol found in various essential oils, induced apoptosis in *Aspergillus flavus* through a mechanism involving ROS accumulation, intracellular Ca^2^⁺ overload, cytochrome c release, and subsequent caspase activation, confirming that oxidative stress is a central mediator of EO-induced fungal cell death.^140^ Furthermore, perillaldehyde induced metacaspase-dependent apoptosis in *Ceratocystis fimbriata* through a cascade of Ca^2^⁺ overload, reactive oxygen species accumulation, and mitochondrial dysfunction, providing direct mechanistic evidence for pro-apoptotic activity of EO constituents against woody pathogens.^141^ In a related study, perillaldehyde triggered metacaspase-dependent apoptotic pathways through cytochrome c release and mitochondrial depolarization in *Aspergillus flavus*, reinforcing the concept that EO-induced oxidative stress can activate programmed cell death pathways even in filamentous fungi.^142^ Lipid peroxidation—the oxidative degradation of polyunsaturated fatty acids within the phospholipid bilayer—is a critical downstream consequence of EO exposure; the reactive lipid peroxides generated amplify oxidative damage,^143^ compromise membrane integrity, and ultimately lead to cellular lysis.^144^ Similarly, benzyl isothiocyanate, a bioactive constituent of cruciferous essential oils, induced oxidative damage in the woody pathogen *Ceratocystis fimbriata* through mitochondrial peroxiredoxin PRX1, highlighting the role of oxidative stress response proteins in EO-mediated antifungal activity.^145^

**Table 1a.** EOs against pathogens of forest and ornamental trees.

Essential-oil source	Pathogen Host	Pathogen Names	Key Findings	References
*Cryptomeria japonica* (various tissues)	Forest trees	*Laetiporus sulphureus*, *Trametes versicolor*, *Rhizoctonia solani*, *Colletotrichum gloeosporioides*, *Fusarium solani*, *Ganoderma australe*	Heartwood oil showed IC₅₀ values of 39–110 µg/mL; dominated by sesquiterpene hydrocarbons (*δ*-cadinene 18.60%, isoledene 12.41%)	Cheng et al. [146]
*Cinnamomum osmophloeum* (leaf chemotypes)	Forest and ornamental trees	*R. solani*, *C. gloeosporioides*, *G. australe*, *F. solani*	Cinnamaldehyde and cinnamaldehyde–cinnamyl acetate chemotypes showed excellent inhibition; Z-cinnamaldehyde most potent	Lee et al. [66]
Thyme (*Thymus vulgaris*), Lavender (*Lavandula angustifolia*), Cypress (*Cupressus sempervirens*)	*Fraxinus angustifolia* seedlings	*Hymenoscyphus fraxineus*	Thyme EO reduced lesion development and improved crown condition in 3-year-old seedlings	Vemić et al. [91]
× *Hesperotropsis leylandii*, *Platycladus orientalis*, *Juniperus communis*	Walnut, oak, cherry laurel	*Phytophthora cactorum*, *P. plurivora*, *P. pseudocitrophthora*, *P. × cambivora*	Significant dose-dependent inhibition; *J. communis* achieved 90.2% inhibition against *P. × cambivora* at 0.5%	Milenković et al. [147]
*Eucalyptus hybrid* (*E. camaldulensis* × *E. tereticornis*)	Forest trees	*G. lucidum*, *T. versicolor*, *Pycnoporous sanguineus*	MIC values 0.5–3.0%; ursolic acid identified as active constituent	Varshney et al. [148]

**Table 1b.** EOs against pathogens of fruit trees and woody crops.

Essential-oil source	Pathogen Host	Pathogen Names	Key Findings	References
*Origanum compactum*, *Thymus satureioides*	Date palm (*Phoenix dactylifera*)	*Fusarium oxysporum* f. sp. *albedinis*	*O. compactum* (MFC 5 µL/mL) and thymol (MFC 0.94 µL/mL) most potent; thymol and carvacrol identified as key constituents	Rahmouni et al. [67]
Chinese cinnamon, Oregano, Holy basil, Thyme, Lemon, Peppermint	Olive (*Olea europaea*)	*Botryosphaeria dothidea*, *Diplodia* spp., *Neofusicoccum parvum*, *Biscogniauxia* spp., *Cytospora pruinosa*, *Nigrospora* spp., *Phaeoacremonium iranianum*	Chinese cinnamon & oregano EOs (plus e-cinnamaldehyde & carvacrol) completely inhibited all 14 fungi; *Do. iberica* and *N. gorlenkoana* most sensitive	Petrović et al. [149]
Clove, Garlic, Tea tree, Rosemary, Thyme, Oregano	Olive (*Olea europaea*)	*Biscogniauxia mediterranea*, *Fusarium* spp., *Verticillium dahliae*, *Lasiodiplodia theobromae*, *Rhizoctonia bataticola*	Clove oil (500–4000 ppm) inhibited *Fusarium* spp., *V. dahliae*, and *L. theobromae*; clove EO demonstrated highest overall efficacy	Krid et al. [72]
Six commercial EOs	Grapevine (*Vitis vinifera*)	*Neofusicoccum parvum* (grapevine trunk disease)	*Thymus capitatus* EO (68.84% carvacrol) achieved complete inhibition at 20 µL; MIC = 250 ppm, MFC = 900 ppm; pure carvacrol MIC = 80 ppm	Trabelsi et al. [74]
*Origanum vulgare* (vapor/fumigation)	Grapevine (*Vitis vinifera*)	*Plasmopara viticola*	24 h post-infection fumigation reduced downy mildew by 95%; primarily acts as plant resistance primer (SA, JA, ethylene pathways)	Rienth et al. [68]
Clove (*Syzygium aromaticum*)	Pistachio (*Pistacia vera*)	*Paecilomyces variotii*	Lowest fungal growth (1.421 cm) at 400 ppm; 400 ppm recommended to reduce growth rate	Yazdanpanah & Mohamadi [150]
Cinnamon, Clove	Apple (*Malus domestica*)	*Botryosphaeria dothidea*, *Colletotrichum gloeosporioides*	EO vapor (60 μL/L) significantly reduced decay incidence; trans-cinnamaldehyde and eugenol bound strongly to CYP51 (in silico)	Wang et al. [151]
*Phoebe bournei* wood EO	Apple (*Malus domestica*)	*Botryosphaeria dothidea*	Controlled apple ring rot in vivo; mechanisms: membrane damage, ROS accumulation, mitochondrial dysfunction; active constituents: (-)-borneol, isoborneol, terpinen-4-ol, guiazulene, o-cymene	Yang et al. [73]
*Heracleum platytaenium*	Apple	*Monilinia* spp., *Venturia inaequalis*	Highest inhibition: 48.8% against *Monilinia* spp., 46.6% against *V. inaequalis*; extraction year influenced activity	Sivrikaya et al. [152]
Thyme, Oregano, Savory, Cinnamon, Mint, Tea tree, Lavender, Eucalyptus, Myrtle	Avocado, Mango, Papaya	*Colletotrichum gloeosporioides*, *Phytophthora palmivora*, *Fusarium solani*, *Botryosphaeria* sp.	Thyme and savory oils achieved 100% inhibition at all tested concentrations (100–2000 µL/L); thymol (73.3%) and carvacrol (71.2%) identified as major components	Sarkhosh et al. [153]
*Cymbopogon martinii*	Papaya (*Carica papaya*)	*Colletotrichum gloeosporioides*, *Fusarium longifundum*	Complete inhibition at 1200–1500 µg/mL; mechanisms: reduced ergosterol, cellular leakage, acidification, ultrastructural damage	Sonigra et al. [76]
*Eugenia uniflora* chemotypes	Papaya	*Colletotrichum gloeosporioides*	IC₅₀ values 0.01–0.09 mg/mL; significant chemical variability among chemotypes; inhibition rates comparable to tebuconazole	Moreira et al. [78]
Thyme, Clove, Cinnamon, Anise, Vitex	Mango (*Mangifera indica*)	*Colletotrichum gloeosporioides*, *Lasiodiplodia theobromae*	Thyme at 5 µL/plate, clove and cinnamon at 8 µL/plate achieved 100% inhibition; thyme fumigation at 66.7 µL/L significantly inhibited postharvest pathogens	Perumal et al. [54]
*Piper* spp. EOs	Mango, Avocado	*Fusarium solani*	*P. lanceaefolium* EO highest inhibition (69.6% at 300 ppm); limonene (78%) and *α*-pinene (74%) contributed substantially	Jaramillo-Colorado et al. [71]
*Dysphania ambrosioides*	Cacao (*Theobroma cacao*)	*Lasiodiplodia theobromae*	Concentrations ≥ 2900 ppm completely inhibited mycelial growth; ascaridole (44.24%), *α*-terpinene (18.88%), o-cymene (14.21%) major components	Yambay et al. [154]
*Cymbopogon citratus*, *Eucalyptus citriodora*, *Ocimum gratissimum*	Cacao (*Theobroma cacao*)	*Phytophthora palmivora*, *Phytophthora megakarya*	*C. citratus* and *O. gratissimum*: 83.80–100% inhibition; reduced leaf susceptibility index from 3.14 to 0.40	Coulibaly et al. [155]
Thymol EO (from *Thymus* spp.)	Goji berry (*Lycium barbarum*)	*Alternaria alternata*, *Botrytis cinerea*, *Cladosporium cladosporioides*	Potent membrane disruption; enhanced antioxidant metabolites and defense-related enzymes; reduced ROS and decay rate	Ali et al. [156]
Cinnamon EO coating (+ slight water loss)	Hardy kiwifruit (*Actinidia arguta*)	*Botrytis cinerea*	Combined treatment reduced decay from 66.7% to 44.7%; activated phenylpropanoid pathway (PAL, 4CL, C4H, SD activities)	Liu et al. [82]
*Artemisia sieversiana*	Pear (*Pyrus pyrifolia*)	*Alternaria alternata*, *Alternaria tenuissima*	MIC = 0.10% (v/v), MFC = 0.12% (v/v); caused hyphal collapse and loss of structural integrity; no toxicity to human cells below 120 µg/mL	Zhang et al. [157]
Cinnamon EO	Peach (*Prunus persica*)	*Rhizopus stolonifer*	0.8 mL/L fumigation significantly reduced soft rot incidence; mechanisms: membrane damage, MDA, H₂O₂, ROS accumulation	Zhao et al. [81]
Thymol, Carvacrol, Linalool, trans-Caryophyllene (from *O. vulgare*)	Peach	*Monilinia laxa*, *M. fructigena*, *M. fructicola*	Thymol and carvacrol showed highest in vitro activity; MIC values: carvacrol 0.02–0.03 µg/µL	Elshafie et al. [61]
Thyme, Oregano, Savory, Rosemary, Marjoram, etc.	Apricot, Nectarine, Plum	*Monilinia laxa*, *Botrytis cinerea*	1% oregano, savory, thyme oils effectively controlled pathogens on apricots and plums, but caused phytotoxicity on nectarines	Lopez-Reyes et al. [77]
*Cuminum cyminum*, *Salvia officinalis*, *Melissa officinalis*	Various fruit trees	*Fusarium oxysporum*, *Rhizoctonia solani*, *Sclerotinia sclerotiorum*, *Verticillium dahliae*, *Pythium* spp., *F. solani*	Dose-dependent inhibition; some oils effective even at low concentrations (50 ppm)	Saraç Sivrikaya et al. [158]
Clove, Cedar wood, Lemongrass, Peppermint, Citronella, Nutmeg	Neem (*Azadirachta indica*)	*Phomopsis azadirachtae*	Citronella and lemongrass oils showed 100% inhibition at 2,500 ppm	Prasad et al. [159]
Rosemary, Wintergreen, Teatree, Patchouli, Palmarosa, Geranium, Eucalyptus	Neem	*Phomopsis azadirachtae*	Wintergreen, Geranium, Palmarosa: 100% inhibition at 2,500 ppm; Palmarosa achieved complete inhibition at only 500 ppm	Nagendra Prasad et al. [160]
Basil, Camphor, Lavender, Rose, Cinnamon	Neem	*Phomopsis azadirachtae*	Basil, camphor, lavender, rose oils completely inhibited growth at 400 ppm; all five inhibited phytotoxin production at 300 ppm	Girish & Fathima [161]

**Table 1c.** EOs against wood-decay fungi and wood preservation.

Essential-oil source	Pathogen Host	Pathogen Names	Key Findings	References
*Artemisia monosperma*, *Cupressus sempervirens*, *Citrus limon*, *Thuja occidentalis*, *Schinus molle*, *Pelargonium graveolens*	Scots pine sapwood	*Hexagonia apiaria*, *Ganoderma lucidum*	*A. monosperma*: EC₅₀ = 31 mg/L (*H. apiaria*) and 53 mg/L (*G. lucidum*); *C. limon* most effective against *H. apiaria*; significant mass loss reduction	Mohareb et al. [69]
Ten EOs (Thyme, Oregano, Sweet flag, Clove, Savory, Birch, Tea tree, etc.)	Beech wood (*Fagus sylvatica*)	*Coniophora puteana* (brown rot), *Trametes versicolor* (white rot), *Aspergillus niger*, *Penicillium brevicompactum*	Thyme, oregano, sweet flag, clove oils showed highest inhibition against molds and *C. puteana*; negligible effect against *T. versicolor*; color changes observed with some oils	Pánek et al. [162]
*Origanum vulgare*, *Cymbopogon citratus*, *Thymus vulgaris*, *Pelargonium graveolens*, *Cinnamomum zeylanicum*, *Eugenia caryophyllata*	Wooden structures	*Trametes hirsuta*, *Laetiporus sulphureus*	*O. vulgare* most toxic (IC₅₀ = 79.1 µg/mL against *T. hirsuta*; 36.9 µg/mL against *L. sulphureus*); carvacrol most potent among major compounds	Xie et al. [163]
*Cymbopogon citratus*, *Cupressus lusitanica*, *Drimys brasiliensis*	*Pinus taeda* (treated specimens)	*Trametes versicolor*, *Pycnoporus sanguineus*, *Gloeophyllum trabeum*	*C. citratus* oil provided best wood protection; classified treated *Pinus taeda* as highly resistant, with least mass loss against *T. versicolor*	de Camargo et al. [164]

**Table 1d.** EOs against postharvest pathogens of woody fruits.

Essential-oil source	Pathogen Host	Pathogen Names	Key Findings	References
Thyme, Clove, Cinnamon, Anise, Vitex	Mango	*Colletotrichum gloeosporioides*, *Lasiodiplodia theobromae*	Thyme at 5 µL/plate, clove and cinnamon at 8 µL/plate achieved 100% inhibition; thyme fumigation at 66.7 µL/L significantly inhibited postharvest pathogens	Perumal et al. [54]
Dill seed EO (DSEO)	Chinese chestnut (*Castanea mollissima*)	*Neofusicoccum parvum*	Dose-dependent inhibition; mechanisms: disruption of cell wall/membrane integrity, oxidative stress, redox imbalance; transcriptomics revealed altered genes related to replication, transcription, translation, lipid/DNA metabolism; in vivo: DSEO and its emulsion effectively prolonged storage life	Liu et al. [70]
Thymol (vapor)	Grapefruit (*Citrus paradisi*)	*Lasiodiplodia theobromae* (stem-end rot), *Penicillium digitatum* (green mold)	5-day exposure to 50 mg/L thymol vapors significantly reduced disease incidence and lesion area; suppressed sporulation; no off-flavor or additional weight loss; GRAS status highlighted	Olmedo et al. [80]
Cinnamon, Clove	Apple	*Botryosphaeria dothidea*, *Colletotrichum gloeosporioides*	EO vapor (60 μL/L) significantly reduced decay incidence; SEM/TEM revealed membrane/organelle damage; transcriptomics showed downregulation of membrane/transport genes; in silico: trans-cinnamaldehyde and eugenol bound strongly to CYP51	Wang et al. [151]
*Phoebe bournei* wood EO	Apple	*Botryosphaeria dothidea*	Controlled apple ring rot in vivo; mechanisms: membrane damage, ROS accumulation, mitochondrial dysfunction; active constituents: (-)-borneol, isoborneol, terpinen-4-ol, guiazulene, o-cymene	Yang et al. [73]
Perilla EO	Chestnut	*Botryosphaeria dothidea*	Dose-dependent inhibition; mechanism: disruption of cell wall and plasma membrane integrity; effectively controlled decay and prolonged storage life	Zeng et al. [165]
Cinnamon EO	Peach	*Rhizopus stolonifer*	0.8 mL/L fumigation significantly reduced soft rot incidence; mechanisms: membrane damage, MDA, H₂O₂, ROS accumulation	Zhao et al. [81]
Thymol, Carvacrol, Linalool, trans-Caryophyllene	Peach	*Monilinia laxa*, *M. fructigena*, *M. fructicola*	Thymol and carvacrol showed highest in vitro activity; MIC values: carvacrol 0.02–0.03 µg/µL	Elshafie et al. [61]
Thyme, Oregano, Savory, Rosemary, etc.	Apricot, Nectarine, Plum	*Monilinia laxa*, *Botrytis cinerea*	1% oregano, savory, thyme oils effectively controlled pathogens on apricots and plums; phytotoxicity on nectarines; cultivar-dependent efficacy	Lopez-Reyes et al. [77]
Thymol EO (from *Thymus* spp.)	Goji berry	*Alternaria alternata*, *Botrytis cinerea*, *Cladosporium cladosporioides*	Potent membrane disruption; enhanced antioxidant metabolites and defense-related enzymes; reduced ROS and decay rate; maintained fruit firmness, color, and quality parameters	Ali et al. [156]
Nanoemulsions (Clove, Garlic, etc.)	Citrus fruits	*Colletotrichum gloeosporioides*, *Neofusicoccum parvum*	Clove and garlic nanoemulsions showed highest in vitro inhibitory activity; significantly reduced lesion development in vivo; phytotoxic effects at higher concentrations	La Quatra et al. [75]
*Artemisia sieversiana*	Pear	*Alternaria alternata*, *Alternaria tenuissima*	MIC = 0.10% (v/v), MFC = 0.12% (v/v); caused hyphal collapse and loss of structural integrity; no toxicity to human cells below 120 µg/mL	Zhang et al. [157]
Cinnamon EO coating (+ slight water loss)	Hardy kiwifruit	*Botrytis cinerea*	Combined treatment reduced decay from 66.7% to 44.7%; activated phenylpropanoid pathway (PAL, 4CL, C4H, SD activities)	Liu et al. [82]

## 6. Conclusion and future perspectives

EOs have considerable potential as multifunctional tools for the sustainable management of fungal and oomycete pathogens affecting woody plants. The evidence synthesized in this review indicates that their activity is governed by a complex interaction among chemical composition, chemotype, pathogen biology, concentration, formulation, host physiology, and environmental conditions. Their multitarget effects—including disruption of cell membranes and walls, interference with ergosterol and cell-wall biosynthesis, mitochondrial dysfunction, oxidative stress, inhibition of spore germination, and, in some cases, activation of host defense responses—provide a strong mechanistic basis for their use as alternatives or complements to conventional fungicides. Nevertheless, compositional variability, volatility, phytotoxicity, limited persistence, and inconsistent translation of laboratory efficacy to plants and field conditions remain major obstacles to practical deployment. Thus, the future development of EO-based disease management should move beyond the identification of a single highly active oil toward standardized, formulation-specific, and pathosystem-tailored technologies.

A major priority is to close the persistent gap between *in vitro* activity and *in vivo* or field performance. Complete inhibition in agar assays frequently requires substantially different doses, exposure conditions, or delivery systems when applied to living plant tissues, reflecting reduced bioavailability, volatilization or degradation, tissue absorption, environmental variability, and host-specific responses. Chemotype variability should therefore be treated as a fundamental determinant of efficacy rather than a secondary source of experimental variation, while phytotoxicity must be evaluated concurrently with antifungal activity because the effective and phytotoxic concentration ranges can differ markedly among host species, tissues, developmental stages, and formulations. Future studies should consequently combine detailed GC–MS/GC–FID chemical profiling with standardized dose–response experiments, including both antifungal efficacy and phytotoxicity thresholds, and should compare free oils with optimized nanoemulsions, encapsulated or controlled-release formulations, and vapor-phase delivery where appropriate. Such approaches would facilitate reproducible chemotype–activity relationships and provide a more reliable basis for selecting candidate EOs for subsequent greenhouse and field evaluation. The importance of this translational approach is supported by the substantial differences already observed between laboratory and plant-based assays and by the improved performance of optimized formulations.

Long-term deployment also requires greater attention to resistance risk, economic feasibility, and field-scale scalability. Although the chemically complex and multitarget nature of EOs may reduce the likelihood of rapid resistance compared with single-site fungicides, repeated exposure—particularly at sublethal concentrations—could still select for pathogens with reduced sensitivity. Resistance monitoring should therefore be incorporated into long-term evaluations through serial-exposure experiments, changes in EC₅₀ values, cross-sensitivity testing, and, where appropriate, genomic and transcriptomic analyzes. At the same time, commercialization will depend on whether EO-based treatments can provide economically viable disease control at orchard, plantation, forestry, wood-preservation, and commercial postharvest scales. Techno-economic assessments should consider raw-material availability, extraction and formulation costs, transport, application frequency, labor, machinery, and disease-control benefits. Locally available EO sources, standardized chemotypes, agro-industrial by-products, targeted delivery, controlled-release systems, and precision application may improve cost-effectiveness by reducing application volumes and unnecessary treatments. Importantly, the most realistic pathway is unlikely to be the complete replacement of synthetic fungicides by EOs alone; rather, EOs may provide their greatest value when strategically integrated with biological control agents, host resistance, reduced-risk fungicides, and other integrated disease-management practices. This approach could simultaneously improve efficacy, diversify modes of action, and reduce chemical inputs.

The next generation of EO research should increasingly exploit integrated omics, chemometrics, artificial intelligence (AI), nanotechnology, and precision agriculture to transform EO development from empirical screening into mechanism-guided and predictive disease management. Transcriptomics, proteomics, metabolomics, lipidomics, and spatially resolved approaches can clarify pathogen targets, stress-response networks, resistance mechanisms, and interactions among EO constituents, while chemical fingerprinting and chemometric analysis can establish reproducible relationships between EO composition and biological activity. These datasets could be integrated with pathogen genomes, host characteristics, environmental variables, EO concentration, and phenotypic responses to develop explainable machine-learning models capable of predicting EO efficacy and identifying synergistic compound combinations. In parallel, nanoemulsions, liposomes, polymeric nanoparticles, nanogels, and controlled-release systems offer opportunities to improve EO stability, dispersibility, adhesion, bioavailability, and persistence, although their cost, environmental fate, phytotoxicity, and regulatory implications require careful assessment. Integration with UAV and satellite imaging, hyperspectral and thermal sensing, IoT-based environmental monitoring, digital disease recognition, variable-rate spraying, and robotic delivery could further enable site-specific application according to disease risk and pathogen hotspots.

Ultimately, successful translation of EOs into woody plant disease management will depend on treating them not simply as natural extracts, but as components of standardized, intelligent, and precision-delivered disease-management platforms. Importantly, efficacy demonstrated in laboratory assays or small-scale postharvest experiments should not be interpreted as evidence of commercial-scale or long-term feasibility. Translation to practical disease management will require pilot- and commercial-scale validation of application systems, including assessment of treatment uniformity, formulation stability, application frequency, operational requirements, cost, environmental fate, and compatibility with existing orchard, forestry, and postharvest infrastructure. Future research should therefore prioritize replicated greenhouse and multi-location field trials, long-term persistence and residue studies, effects on beneficial microorganisms and pollinators, environmental fate, compatibility with integrated pest management, and standardized reporting of EO origin, extraction method, chemical profile, formulation, dose, application mode, and environmental conditions. Integration of chemical standardization with omics, AI-assisted prediction, advanced formulation, digital phenotyping, and precision application could substantially improve the predictability, scalability, and sustainability of EO-based disease control. Rather than replacing synthetic fungicides in isolation, this integrated strategy offers a more realistic pathway for reducing dependence on conventional chemicals while maintaining effective disease suppression and improving the resilience and environmental compatibility of woody plant production and forest health.

## Data Availability

All datasets generated and analyzed during this study are included in this published article.
